# Four-corner arthrodesis of the wrist using Variable Angle Locking Compression Intercarpal Fusion Plate (VA LCP ICF Plate; Synthes^®^): pre- and postoperative radiological analysis and clinical outcome in long-term evaluation

**DOI:** 10.3205/iprs000141

**Published:** 2019-11-15

**Authors:** Christian Eder, Ariane Scheller, Nina Schwab, Björn Dirk Krapohl

**Affiliations:** 1 Centre for Musculoskeletal Surgery, Charité – Medical University, Campus Virchow Clinic, Berlin, Germany; 2 Healthcare Center Meviva, Berlin, Germany; 3 Carl-Thiem-Klinikum, Cottbus, Germany

**Keywords:** wrist fusion, SNAC wrist, SLAC wrist, intercarpal arthrodesis

## Abstract

Long persisting scaphoid non-unions or scapholunate ligament ruptures can lead to carpal collapse.

The resulting clinical symptoms are restrictions in the range of motion, pain, and loss of grip strength. The symptomatic treatment so far offers different options. In our study, the Variable Angle Locking Compression Intercarpal Fusion Plate (VA LCP ICF Plate) by Synthes^®^ was used in 11 cases of advanced carpal collapse for a four-corner fusion of the wrist. The intra- and postoperative as well as follow-up results have been assessed and compared with those of current literature.

The results of the Manchester-Modified Disability of the Shoulder, Arm and Hand Score (M²-Dash) showed an average of 41.5 points (MD=44/SD=16.62/MIN=21/MAX=65).

One of the re-evaluated patients complained about pain at rest. One patient stated pain after mild strain; 4 patients complained pain after heavy burden (e.g. boxing, weight lifting).

Measuring the range of motion, the operated hand showed a maximum in dorsal extension of 78.31% and in flexion of 57.89% compared to the contralateral, non-operated hand. In performance testing the fist clenching sign as well as pinch grip were complete and void of pain in 100%, whereas opposition (dig. man. I to V) was complete in five patients (83.33%), with moderate pain in one patient (16.67%) and a persisting gap of 0.2 cm in n=1 (16.67%). In comparison with the current literature regarding healing rates, complications, and follow-up results, we recommend the Synthes^®^ VA LCP ICF Plate as a good surgical option in patients suffering from advanced carpal collapse.

## Zusammenfassung

Lang bestehende Skaphoidpseudarthrosen oder skapholunäre Bandrupturen können zu einem karpalen Kollaps führen. Symptome dessen sind unter anderem Bewegungseinschränkung, belastungsabhängige Schmerzen und Kraftreduktion. In der aktuellen Literatur sind diverse unterschiedliche Prozeduren und therapeutische Optionen für dieses Krankheitsbild beschrieben. In der vorliegenden Studie werden das intraoperative Handling und die postoperativen klinischen und radiologischen Ergebnisse der Variable Angle Locking Compression Intercarpal Fusion Plate (VA LCP ICF Plate, Firma Synthes^®^) für die mediokarpale Teilarthrodese (four-corner fusion) mit den Ergebnissen anderer Verfahren und Implantate der aktuellen Literatur verglichen. Das Implantat wurde bei 11 Patienten verwendet; die Nachuntersuchung ergab folgende Ergebnisse: Manchester-Modified Disability of the Shoulder, Arm and Hand-Score (M^2^-DASH) mit einem Durchschnittswert von 41,5 Punkten (MD=44/SD=16,62/MIN=21/MAX=65). Ein Patient klagte über Ruheschmerz, ein weiterer Patient über Schmerz nach leichter Beanspruchung der Hand, wohingegen vier Patienten Schmerzen nach stärkerer Belastung (beispielsweise Boxen, Gewichtheben) beklagten. Die Bewegungsprüfung zeigte folgende Resultate: Dorsalextension mit einem Durchschnittswert von 78,31% und Palmarflexion mit einem Durchschnittswert von 57,89% (jeweils der operierten Hand im Vergleich mit der nicht verletzten Hand). Der Faustschluss war bei allen Patienten unbeeinträchtigt, die Kleinfinger-Daumen-Oppositionsprobe ließ sich bei 83,33% der Patienten unbeeinträchtigt feststellen. Ein Patient beklagte bei Letzterem das Auftreten von Schmerzen, bei einem weiteren zeigte sich eine persistente Distanz von 0,2 cm (je 16,67%). Im Vergleich mit der aktuellen Fachliteratur bezüglich Heilungsraten, Komplikationen und Nachuntersuchungsergebnissen kann die VA LCP ICF Platte als gute Option der mediokarpalen Teilarthrodese empfohlen werden.

## Introduction

Persisting scaphoid non-unions and scapholunate ligament ruptures are the main causes for developing an insufficiency in carpal joint structure (carpal collapse, named as scaphoid nonunion advanced collapse = SNAC wrist or scapholunate dissociation advanced collapse = SLAC wrist respectively) [1], [2], [3], [4], [5], [6], [7], [8], [9], [10], [11], [12], [13], [14], [15], [16]. Watson and Ryu classified the severity of degenerative changes into three stages as shown in Table 1 (Tab. 1) [17].

The specific therapeutic surgical options rely on this classification, displaying the here shown possibilities:

- stage I: scaphoid reconstruction and osteosynthesis (for SNAC) or scapholunate ligament refixation (for SLAC), both combined with resection of the radial styloid (styloidectomy)
- stage II: proximal row carpectomy (PCR) or midcarpal arthrodesis (e.g. three- or four-corner fusion)
- stage III: midcarpal arthrodesis including excision of scaphoid bone (with or without bone graft)

**Figure 1:**
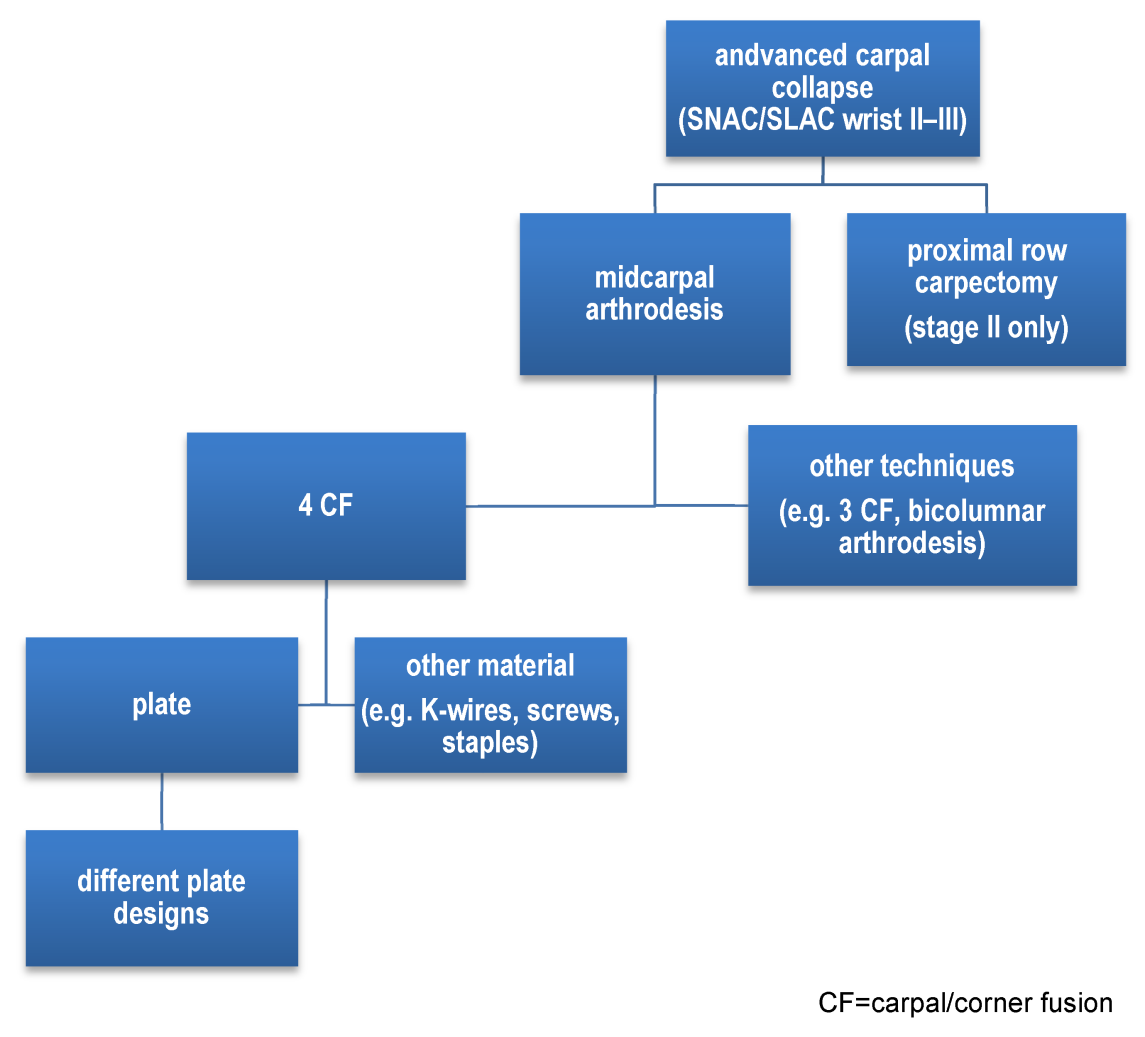
Short algorithm for treatment options of advanced carpal collapse (stages two and three) [1], [3], [4], [9], [10], [11], [13], [14], [15], [16], [18], [19], [20], [21], [22], [23], [24], [25], [26], [27], [31], [32], [33], [34], [35], [42], [43], [45], [46], [47], [50]

Simplified grouping of operative approaches in stage II and stage III carpal collapse leading to specific study topic (Figure 1 (Fig. 1)).

The four-corner fusion, primarily described by Watson and Ryu, is one of the most popular and common procedures for treating stage II or stage III carpal collapse [17]. However, there is disagreement concerning the best osteosynthesis material for this surgery [3], [16], [18], [19], [20], [21], [22], [23], [24], [25], [26]. While traditionally Kirschner wires, staples or compression screws have been used, in 1999 circular plates were introduced, promising better results [27]. Nowadays, many different plate designs are available on the market, but long-term results are currently scarce.

Therefore, the purpose of this study is to evaluate the long-term clinical outcome after four-corner arthrodesis of the wrist using the Variable Angle Locking Compression Intercarpal Fusion Plate by Synthes^®^.

## Material and methods

Between 2011 and 2013, 11 patients underwent surgical treatment including implantation of a Variable Angle Locking Compression Intercarpal Fusion Plate (Synthes^®^) in the Centre for Musculosceletal Surgery of the Charité, Medical University of Berlin. All patients were explored clinically and radiologically (either X-ray or additionally computertomographic or magnetic resonance imaging if medically advised) for diagnosis and preoperative planning.

**Figure 2:**
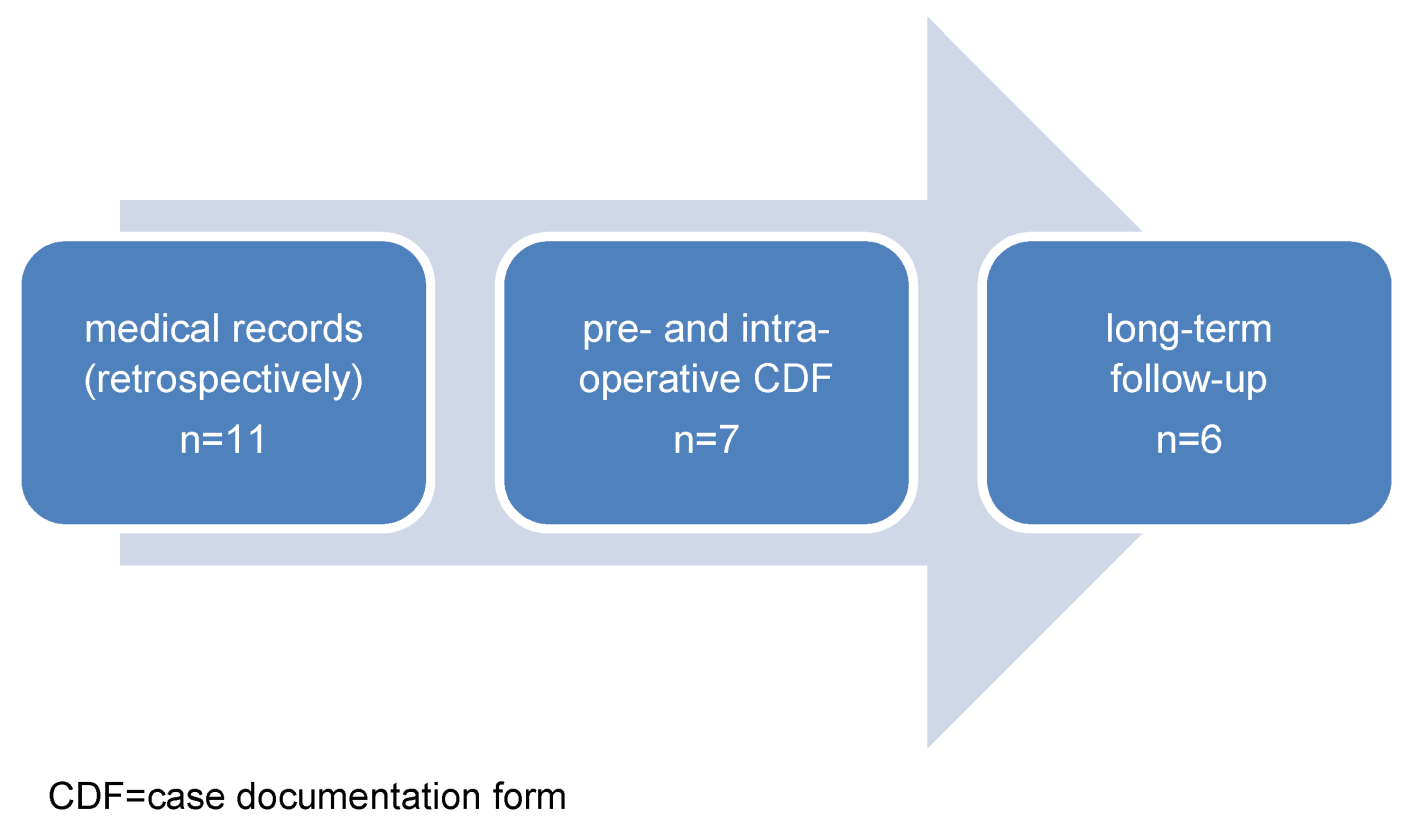
Data collection during patients’ clinical process

To present convincing data, the patients included in the study were interviewed and examined during different steps of the diagnostic and therapeutic process as shown in Figure 2 (Fig. 2).

Retrospectively, patients’ medical records have been reviewed. Therefore, age and gender, previous medical history, trauma history, operation technique (approaches, operation time, complications), pre- and postoperative radiological images, outcome up to three months after the surgery, and fit of the implant were analyzed and summarized under short-term evaluation of the explained modified treatment. 

Additionally, 7 patients agreed in ascertaining data in form of a pre- and intraoperative case documentation (case documentation form 1, CDF 1).

This CDF 1 was presented by Synthes^®^ and was used in the regular treatment of the individual in-patient’s stay to get a detailed view on specific interests (such as carpal height pre- and postoperatively). In this study, the data have been assessed and compared retrospectively. 

Final and core part of this study is long-term evaluation. Patients under 18 years and those who were unable to give informed consent were excluded according to guidance of the local ethics committee. Due to this constraint and patient’s non-response, in conclusion, a total number of 6 patients were re-evaluated. This long-term evaluation consisted of obtaining a general health questionnaire (therefore a shortened SF-36 was chosen) [28] and the Manchester-Modified Disability of the Arm, Shoulder and Hand-Score [29]. Based on a standardized protocol including range of motion, sensory deficiencies, pain, and strength, all patients underwent a clinical examination by a single surgeon. Grip strength was evaluated by using the hand-held dynamometer (JAMAR^®^) [30]. 

Generating radiological images in long-term evaluation was relinquished – due to no or just moderate clinical conspicuousness; the radiation exposure would not have been ethically and medically justifiable. 

All descriptive statistics for our approximately normally distributed data were performed using average/mean (MD), standard deviation (SD), minimum (MIN) and maximum (MAX) and percentage. SPSS v24.0 (SPSS Inc., Chicago, Illinois) has been used to create statistics. 

Based on the Medline database a literature research provided the data for comparing the findings of this study with those of other working groups. 

The study protocol was approved by the local ethic boards. Written consent was obtained from all patients.

## Results

### Patients’ data

Eleven patients underwent implantation of Synthes’ Variable Angle Locking-Compression Intercarpal Fusion Plate (VA LCP ICF Plate). 6 of them (54.55%) were male, whereas 5 patients were female gender (45.45%). The average age was 53.27 years (MD=45/SD=16.38) with a maximum of 74 and a minimum of 25 years. 

Indications for implantation of VA LCP ICF Plate (total number: n=11) were:

- scapholunate advanced collapse (SLAC) n=4
  - stage II (1 case)
  - stage III (3 cases)
- scaphoid nonunion advanced collapse (SNAC) n=5
  - stage II (3 cases)
  - stage III (2 cases)
- de Quervain fracture with instability, initial treatment in a different hospital (1 case)
- carpal instability non-dissociative (CIND) caused by avascular necrosis of the lunate (Kienböck’s disease) stage IIIa (1 case)

All of these 11 patients underwent standard operation procedure for a four-corner arthrodesis, always performed by the leader of the clinic’s department for hand surgery. 6 of these patients (54.55%) got a six hole VA LCP ICF Plate; due to a better fit, assessed during the operation, 5 patients were treated with a seven hole variant of the Synthes^®^ plate. Additionally, 10 patients got their scaphoid bone excised. In these cases, cancellous bone of the scaphoid was extracted and reimplanted. Therefore, no further spongiosa plasty from the iliac crest was necessary. 

The scaphoid bone remained in situ in one case (carpal instability after avascular necrosis of the lunate) and hence an autologous bone graft transfer from the iliac crest was performed to achieve a satisfactory implant fit and to improve further healing rates. 

Following our experiences and recommendation of current literature, immobilization (forearm cast with thumb spica) for 4 to 6 weeks was implemented in postoperative care [20]. 

For long-term re-evaluation two patients were lost in follow-up and three patients did not want to participate. In conclusion, six patients were clinically re-examined with a mean follow-up of 37.67 months (MD=35.5/SD=7.69/MIN=30/MAX=49). The group of patients, who underwent long-term clinical examination showed the following characteristics:

- 50% male and 50% female patients
- average age of 57.83 years
- comorbidities: 1 patient suffering from nerve lesion of the upper extremity after trauma, 1 patient with total shoulder-replacement and depressive disorder

### Short-term evaluation

Concerning the short-term evaluation, data of 11 patients were analyzed. Post-operative immobilization time of 4 to 6 weeks was conducted. The decision was based on intraoperative stability of the arthrodesis and patient’s compliance in participating in postoperative treatment. 

After about three months after finishing physiotherapy all patients presented with incipient bone healing and good siting of the implant based on the last x-ray images (or CT if plain radiograph findings were suspicious). Except of one case where there was a broken screw, there were no further complications. The screw breakage may be related to the patient’s incompliance in postoperative treatment (no immobilization was tolerated since he did weight lifting shortly after surgery). 

### CDF 1

Seven patients agreed in taking further pre- and intraoperative data in this standardized form (“Pre- and intraoperative Case Documentation Form” by Synthes^®^). 

The main goal of this form was to generate more detailed information about the specific radiographic characteristics for each patient individually:

- dorsiflexion deformity of the lunate
- carpal height (Youm)
- ulnar wrist translation (Chamay)

Furthermore, it was designed to explore the surgeons experience regarding the implant during the procedure. We evaluated the subitems concerning the special Synthes^®^ reaming guide for the VA LCP ICF Plate “fit”, “handling” and “holding strength” with good. Furthermore, the “ease of reaming”, “reaming depth”, “plate placement” and “assessment of quality of reduction” were considered good on a scale reaching from excellent to poor (excellent – good – satisfactory – poor). The “assessment of final stability” resulted in four excellent intraoperative findings and three good ones. Just the “ease of drilling VA-locking screws through reaming guide” was judged in 3 cases with satisfactory only. Caution is advised to prevent screw breakages intraoperatively. 

#### Preoperative x-ray evaluation

One patient brought pre-operative x-ray images from an external hospital; due to technical incompatibility the preoperative radiographic analysis could not have been done properly; therefore, we included just 6 patients. 

The dorsiflexion of the wrist in x-ray analysis intraoperatively imposed with an average of 42.83° (MD=46/SD=15.37/MIN=20/MAX=60). 

**Figure 3:**
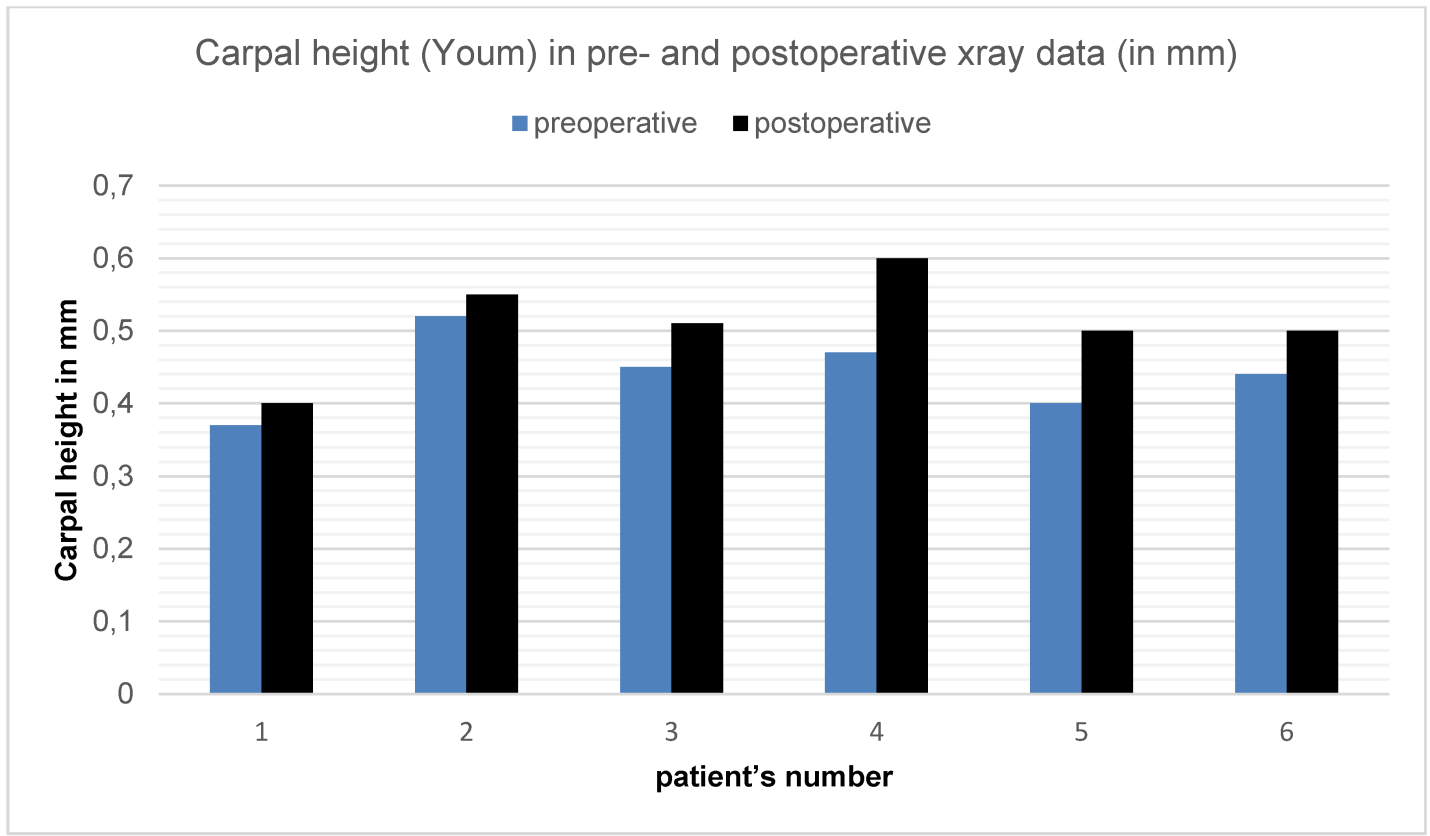
Carpal height (Youm) measured in pre- and postoperative x-ray data of six long-term follow-up patients

Measuring the Carpal height (Youm) shows an average of 0.44 cm (MD=0.45/SD=0.05/MIN=0,37/MAX = 0.52) (Figure 3 (Fig. 3)).

The average of the ulnar translation of the wrist [position of the lunate] (Chamay) is 0.30 cm (MD=0.31/SD=0.04/MIN=0.23/MAX=0.34).

#### Postoperative x-ray evaluation

The comparison of pre- and postoperative carpal height (Youm) is shown in Figure 3 (Fig. 3).

### Long-term evaluation

Six patients were included. In four patients (66.67%) the carpal collapse affected the dominant hand (right hand always) and in 2 cases (33.33%) the non-dominant hand (left hand always). 

The results in the shortened SF-36 general health survey (self-reporting health status for the last four weeks) showed no significant restrictions. Asking for the general health status (independent from age and further comorbidities) in self-estimation two patients answered “good”, four said “satisfying”. No further significant results could be found in analyzing the SF-36.

The outcome of the Manchester-Modified Disability of the Shoulder, Arm and Hand-Score (M²-Dash) is an average of 41.5 points (MD=44/SD=16.62/MIN=21/MAX=65). 

One of the re-evaluated patients complained about pain at rest. One patient stated pain after mild strain; 4 patients mentioned pain after heavy burden (e.g. boxing, weight lifting).

Furthermore, three patients noticed dysaesthesia especially in digiti manus IV and V dorsal. In one case, dysaesthesia was already known before surgery. 

In exploring the range of motion of the operated hand, the following results can be presented:

- dorsal extension: average 54.17° (MD=52.5°/SD=19.08°/MIN=30°/MAX=80°)
- flexion: average 45.83° (MD=47.5°/SD=23.54°/MIN=5°/MAX=75°)
- ulnar abduction: average 26.67° (MD=30°/SD=14.02°/MIN=10°/MAX=40°)
- radial abduction: average 24.17° (MD=27.5°/SD=8.01°/MIN=10°/MAX=30°)

The differences in mobility of the wrist between the operated and the other hand are shown here (percentage ROM of operated hand compared to non-operated hand):

- dorsal extension: 78.31% of non-operated hand
- flexion: 57.89% of non-operated hand

Additionally, a performance test was conducted:

- fist clenching: complete without pain in 100%
- pinch grip: complete without pain in 100%
- thumb opposition: complete in n=5 (83.33%), moderate pain n=1 (16.67%), persisting gap of 0.2 cm in n=1 (16.67%)

**Figure 4:**
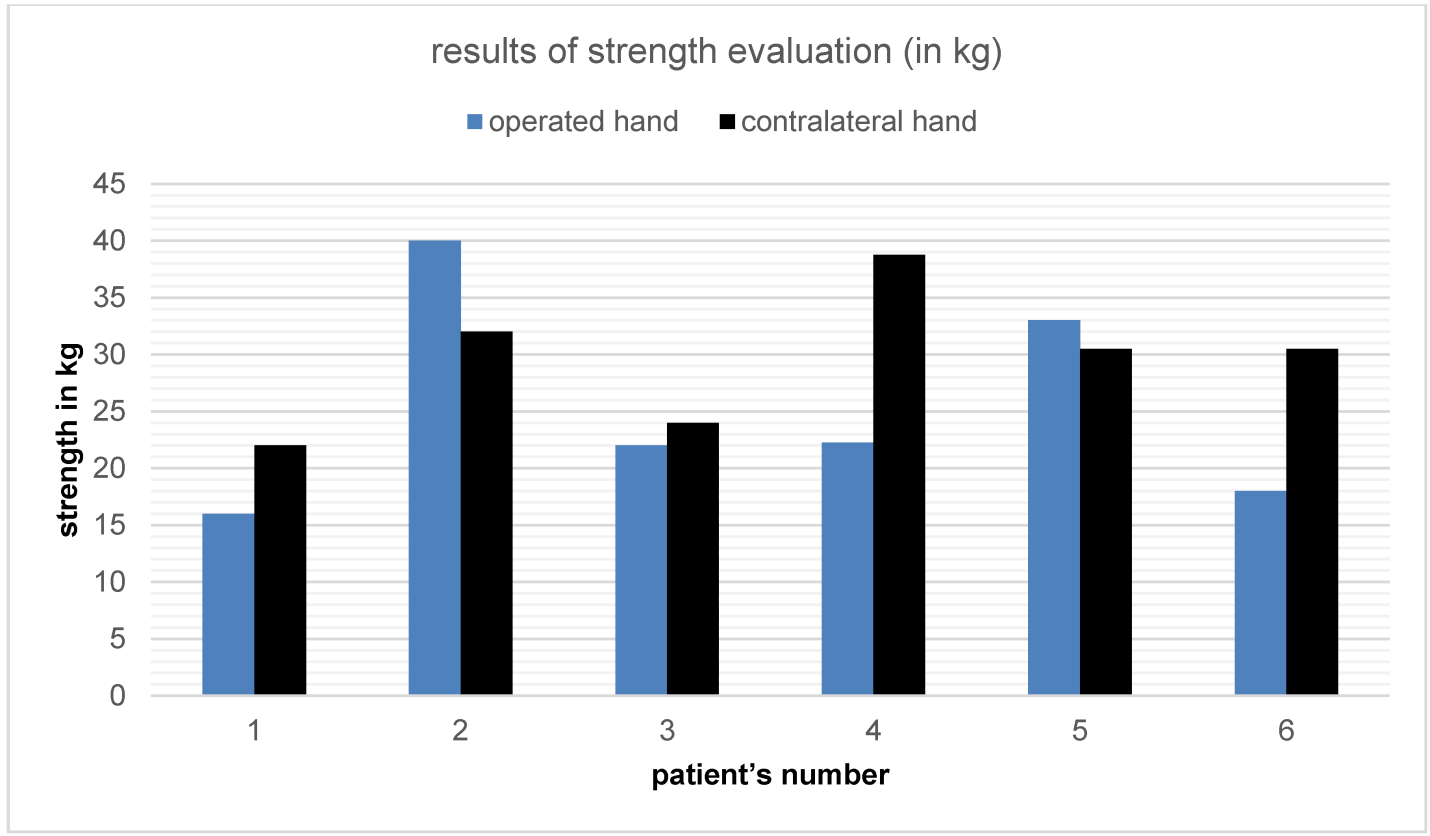
Results of grip strength evaluation of operated and contralateral hand by using the hand-held dynamometer (Jamar^®^) in six long-term follow-up patients

Grip strength evaluation was performed by using the hand-held dynamometer (Jamar^®^). Figure 4 (Fig. 4) shows the results comparing reached maximum grip strength of operated hand versus non-operated hand (highest level out of three tries). 

**Figure 5:**
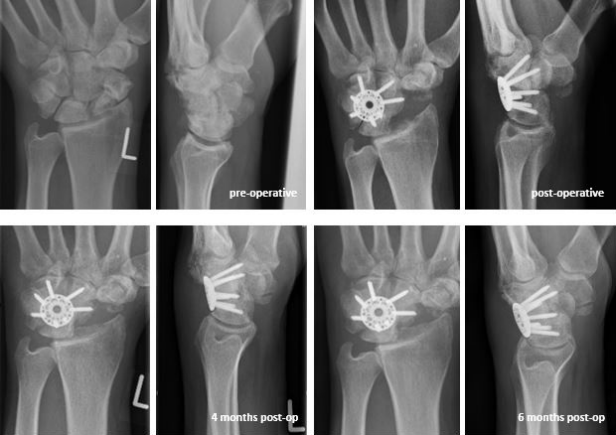
X-ray series of an advanced carpal collapse (SNAC wrist II–III) treated with Synthes^®^ VA LCP ICF Plate up to six months after operation

Figure 5 (Fig. 5) shows an X-ray series of an advanced carpal collapse (SNAC wrist II–III) treated with Synthes^®^ VA LCP ICF Plate up to six months after operation.

## Discussion

There is a variety of different operation techniques, treating carpal instabilities like SNAC or SLAC wrist deformities in stage II or III. Most commonly and best described in literature are the 4-corner fusion of the wrist and the proximal row carpectomy [1], [2], [3], [4], [5], [6], [7], [9], [10], [11], [13], [16], [18], [19], [31], [32], [33], [34], [35]. Nonetheless, some authors recommend the 3-corner fusion, bicolumnar intercarpal arthrodesis or other modifications as better options for treating wrist instabilities [14], [15]. 

Based on a systematic literature review we want to discuss and compare the 4-corner fusion, with emphasis on the variable angle locking plate (VA LCP ICF Plate by Synthes^®^), with other well accepted treatment options. Furthermore, we want to find out whether there were any differences between the Synthes^®^’ Variable Angle Locking Compression Intercarpal Fusion Plate and other fixation techniques used for the wrist arthrodesis (either traditional fixation techniques or different plate designs).

Proximal row carpectomy (PCR) and midcarpal arthrodesis (MCA) are motion preserving options for the treatment of stage II carpal collapse [9]. Many studies have been published to discuss the outcome of both operations. Exemplarily, Dacho et al. stated, that the PCR is easier to perform than the MCA operation [9]. However, the biomechanical situation after excision of the proximal carpal row is more dysfunctional due to the creation of incongruent articular surfaces [4], [9]. Furthermore, Brinkhorst et al. explored better functional outcomes (according to the Sollerman hand function test) of PCR patients comparing to those with MCA [36]. Additionally, there were specific complications for PCR and MCA mentioned in literature: On the one hand PCR can cause arthrosis in the radiocapitate region (particularly with necessity for denervation procedures), whereas on the other hand MCA may lead to insufficient cartilage removal, persisting pain, lower functional level postoperative, improper realignment of carpal height with nonunion in consequence (particularly with necessity for complete arthrodesis with loss of motion preserving keynote) [2], [4], [5], [9], [10], [11]. 

Williams et al. described significantly higher rates of secondary operations after 4 CF compared to PRC procedures due to non-unions, hardware impingement and others. They saw no significant differences in conversion rate to total wrist arthrodesis in the PRC group versus 4 CF group patients [37]. In overall conclusion of a randomized clinical trial comparing proximal row carpectomy and four-corner fusion, Aita et al. resumed no significant statistical differences in clinical and functional results between both salvage procedures [38].

In systematically researching current literature, it becomes obvious that there is a variety of studies seeing advantages and disadvantages for both procedures with consequently recommending either PCR or MCA [34] or seeing no statistical significant differences [38].

Therefore Mulford et al. performed a systematic analysis for comparing both operative treatments for stage II carpal collapse. Although there were no significant differences in pain relief, grip strength, subjective outcomes (such as patient’s satisfaction) or reaching the state of complete arthrodesis, the authors stated a higher rate of radiocapitate arthrosis after PCR and 10% higher rate of general complications like non-union, dorsal impingement and material conflicts after MCA [34]. The post-operative flexion-extension arc was 10 degrees lower after MCA in comparison to PCR operation. In conclusion of Mulford et al.’s systematic review there is no clear overall difference between MCA and PCR whereby both procedures are equal options in therapeutic considerations [34].

The other systematical review comparing four-corner fusion (4 CF) and PCR by the working of group of Saltzman et al. stated a significant higher postoperative radial abduction range after MCA, whereas dorsal extension and palmar flexion as well as ulnar abduction displayed no significant differences [39]. Furthermore, the evaluated grip strength after 4 CF showed higher levels than the PCR cohort. Patient’s satisfaction and pain severity did not lead to any statistically significant differences [39]. 

There are many well described variations of midcarpal arthrodesis. Common options are 3-corner arthrodesis and 4-corner arthrodesis as well as bicolumnar arthrodesis of the wrist [13], [14], [15]. Klausmeyer et al. documented a more physiological situation after performing 3-corner arthrodesis due to better articulating surfaces [15]. However, Draeger et al. showed a restricted range of motion in wrists after bicolumnar arthrodesis [14]. They supposed a higher fusion rate because of leaving greater bone surfaces due to not excising the scaphoid bone [14].

Therefore, no clear recommendation for either one or another varied midcarpal arthrodesis operation is given so far. In our study, all patients were treated with a 4 CF. 

Following the earlier presented decision tree for treatment of stage II and III carpal collapses, the next step is to consider the right material for the midcarpal arthrodesis operation technique. 

After the different variants of traditional techniques (e.g. staples, screws, K-wires), initially the NLDP (e.g. the Spider™ Limited Wrist Fusion Plate, Kinetikos Medical Inc., San Diego, CA, USA) were designed as a new fixation system for 4 CF [4].

In literature research, a lot of case reports or series with either using traditional ostesynthesis material or NLDP are to be found. Comparing studies are rare. 

One of these is the study published by Mavrogenis et al. They compared the long-term results of patients with 4 CF with either K-wires, headless compressive screws or a circular plate [40]. They reported patient’s satisfaction and improvement of pre-operative pain level, range of motion and grip strength in all groups. Additionally, they displayed a fusion rate of 90.3% and a partial fusion in 9.7%. The partial fused wrists were treated in two cases with K-wire MCA and in one case with a circular plate. The overall complication rate is given with 10% of total: 2 patients with the circular plate (1 case of impingement and 1 case of regional pain syndrome) and one patient after K-wire/ headless compression screws fixation (impingement) [40]. 

Vance et al. compared the results after 4 CF with non-locking dorsal plates (NLDP) in one group with 4 CF done with traditionally used materials (wires, staples or screws) [23]. The study group discovered non-union rates of 3% in traditional fixation techniques against 26% in the NLDP group [19]. Additionally, they reported impingement rates of 3% in the traditional group versus 22% in the plate group [19]. Grip strength, range of motion and Disability of Arm and Shoulder (DASH) score also showed less satisfactory results in the NLDP group [19]. Furthermore, Chung et al. also reported that even pain relief was poorer after 4 CF with non-locking plate [4]. They have seen no significant pain decrease but an overall increasing patients’ satisfaction after the operation. Only two patients described complete pain-freeness. As conclusion, Chung et al. claimed further investigations due to not full satisfying outcomes [4]. 

Opposingly, the operation technique using a plate for performing the four corner arthrodesis has its advantages: less infections (especially pin infections after K-wire implantation), absence of implant protrusion, no need for further operations (like implant excision when K-wires have been used) [3], [19], [31].

The study group of Hernekamp et al. reported of no statistical differences comparing patients with K-wire 4 CF and locking plate 4 CF [41].

Pauchard et al. stated that the final decision about the most adequate material for performing midcarpal arthrodesis has not been made yet [20]. Therefore, different techniques are currently used. Inconsistent arguments can be found in literature whether screws, K-wires, staples or plates are the best material to reach the most satisfying results in operating on carpal collapses [19], [20]. 

Evaluations of the non-union rates after using NLDP when performing a 4 CF of the wrist show different results. Kendall et al. stated a high rate of insufficient bony fusion with 62.5% [42] whereas Merrell et al. reached a 100% union rate after using an internal plate fixation technique [22]. Different authors reported non-union rates after 4 CF with traditional fixation materials ranging from 0% [43], [44] up to 17% [45], [46]. Shindle et al. presented a non-union rate of 25% and an overall complication rate of 56% after performing 4 CF via internal plate fixation [47].

In our study we used autogenous bone graft taken from the excised scaphoid bone in 10 cases and reached a 100% union rate. Which may have contributed to the good outcome in healing was the included reaming guide, leading to a better placement of the material. Furthermore, the rigid system with locking screws may contribute to bony fusion, as evaluated by Tielemans et al. using the same implant with reaching bony fusion in 100% as well [48] Drác et al. reported of no non-union after 4 CF with VA LCP ICF Plate in their study population (abstract only, article in Czech) [35]. 

Furthermore, Friedel et al. stated the necrosis of the lunate bone to be a strict contraindication for implanting a plate for 4 CF [18]. We had one case with carpal collapse due to Kienböck’s disease and can report good outcomes after implanting the VA LCP ICF Plate. 

Another important and objective differentiator is the rate of complications occurring after 4 CF with either traditional fixation material or NLDP in different designs. 

Chung et al. reported of three cases with screw break postoperatively (complication rate of 27.3%) [4]. 

The working group of Merrell, using a second-generation circular plate, displayed 2 cases of material failure including 1 case of broken plate and 1 case of screw back-out (total of 28 patients) [22]. 

Mantovani et al. performed an outcome study after 4 CF with a circular titanium plate with 4 non-locking screws (Carpal Button, SBi Interntional, Peronnas, France). They achieved complete fusion in 18 of 19 patients (94.7%) [24]. Additionally they reported of one case of screw break and two cases of persisting pain. This plate design only allows positioning one screw in each carpal bone. Mantovani et al. described this as a disadvantage because intraoperative screw break-outs are not easy to correct and compensate [24]. The necessity for drilling two screws in each bone was stated by other study groups as well [25]. The VA LCP ICF Plate by Synthes^®^ (six or seven hole design) used in this study allowed the performing surgeon to consider the individual anatomic differences and decide in each case how many screws to be used to achieve a stable result. 

Due to these high complication rates and a high risk of non-union, further investigations have been pursued. As consequence, the NLDP were ousted by the new generation of internal fixation method: LDP (locking dorsal plates) in different designs and variations (like the here used Variable Angle Locking Intercarpal Fusion Plate by Synthes^®^) [13], [21]. 

Reissner et al. published their comparison of 4 CF patients either treated with a non-locking plate (Spider plate, Integra LifeSciences.Corp., Plainsboro, NJ) or with a dorsal locking fusion system (Flower plate, KLS Martin group, Tuttlingen, Germany) [49]. In overall conclusion, they recommend the locking plates due to their lower rates of dorsal impingement (5% for locking plates versus 30% for non-locking plates) and lower rates of material loosening [49]. 

Rudnick et al. presented a fusion rate of 80% by using the Xpode^®^ Cup as variant of a LDP (total of 26 operated wrists in 24 patients) [21]. They reported of one case in which a complete wrist arthrodesis had to be performed due to persisting pain and the development of radiolunate arthritis. There were also two cases of screw break-out [21]. Furthermore, five patients imposed with necessity for further surgical treatment because of dorsal adhesion of joint capsule, persisting pain within material conflict and/or impingement symptoms [21]. 

Luegmair et al., using the same implant, remarked a union rate of 92% [50]. Whereas Rhee et al. reported of 96% of bony healing after using the Xpode^®^ Cup [25]. 

Chaudhry et al. published their findings after perfoming 4 CF with the Aptus^®^ LDP by Medartis, showing 2 cases of initial non-union with necessity for revision [51]. 

The working group of Woehl et al. described bony fusion in all patients (n=11) receiving 4 CF with the Aptus^®^ plate (Medartis, Basel, Switzerland). They reported of two complications: one screw malpositioning in the pisotriquetral joint and one proximal positioning of implanted plate leading to restricted mobility and pain after stressful movement [52]. As advantage of the Aptus^®^ plate compared to the here used Synthes^®^ implant they described a lower profile, the smaller diameter and more screw options [52]. But in conclusion they reported of more difficulties in plate positioning due to the missing reaming guide. We can underline this argument due to our good experiences illustrated in the CDF 1 results. 

The overall comparison of outcomes in grip strength and range of motion postoperatively is quite difficult, because specific data is commonly missing in the presented studies. If the articles include details about the postoperative clinical evaluation, there is no standardization making direct comparison, even statistically, impossible. Table 2 (Tab. 2) therefore is just a schedule with included references, using NLDPs or LDPs and giving information about the long-term clinical results.

Tielemans et al. presented their functional outcome score (QuickDASH) after 4 CF using the VA LCP ICF Plate to be improved and the pain level to be decreased post-surgery. They reported of 10% dorsal impingement with necessity to plate removal and 14% of all patients suffering from complex regional pain syndrome [48].

In comparison to studies dealing with different techniques, material and plate designs respectively, the ICF plate by Synthes^®^ seems to be a good option for treating carpal collapse, which we illustrated with our findings as well. 

In the long-term evaluation there were no complications like non-union of bony reconstruction, impingement or further material failures in our study population. The examined range of motion and grip strength were comparable to the results of other studies dealing with different implant variations. 

As limitation of our study, only few patients could have been long-term re-evaluated. Additionally, there was no comparison group with other implants in our hospital; therefore, we could draw a parallel with other study results only. A larger multicenter study could solve these problems. A randomized clinical trial with different plate designs to afford more comparable results would be desirable. Another disadvantage is the lack of direct and statistical comparison of the results presented in current literature due to the non-standardized dissemination and elicitation of examination data. Thus, it was decided within this study to present all data as descriptive data.

Advantageously, all operations were performed by the same surgeon. Additionally, the long-term re-evaluation was always done by the same physician to reach the highest level in commensurability. We used only evaluated and well-proofed questionnaires and diagnostic measures to survey patient’s state of health.

## Conclusion

The four-corner fusion is a salvage operation for advanced carpal collapse such as SNAC and SLAC wrist. K-wires were the gold standard for the operation procedure formerly. By using K-wires, good consolidation rates were achieved. Disadvantageously, higher infection rates, K-wire dislocations and tendon ruptures are described in the literature. 

Comparing the used locking plate by Synthes^®^ with the K-wire osteosynthesis, the plate showed similar consolidation rates with less complications. Advantageously, no second operation for K-wire removal is needed. Furthermore, the evaluated range of motion, especially in long-term follow-up, is remarkably higher in plate arthrodesis. 

The non-locking plates, being the ancestor plate implants for four-corner arthrodesis, also showed complications like plate dislocations, missed healing and material failure, which were less frequent in the newer dorsal locking plate designs.

In overall comparison, Synthes^®^ VA LCP ICF Plate is a more expensive option than K-wires or NLDPs for four-corner fusions, but our long-term evaluations showed comparable healing rates and distinctly better postoperative results especially in grip strength and range of motion.

## Notes

### Competing interests

The authors declare that they have no competing interests.

## Figures and Tables

**Table 1:**
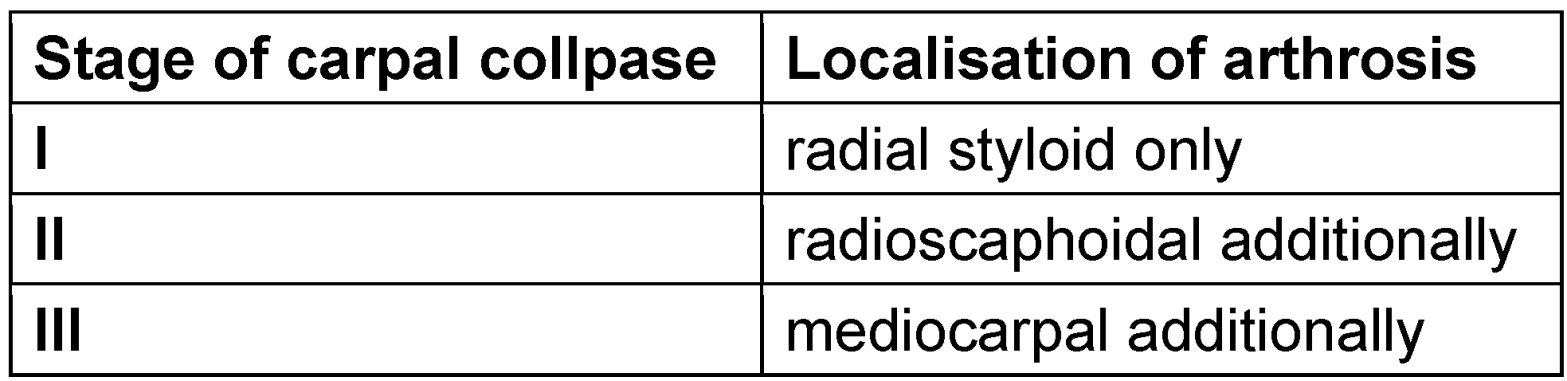
Stages of advanced carpal collapse according to x-ray related visible arthrosis according to Watson and Ryu [17]

**Table 2:**
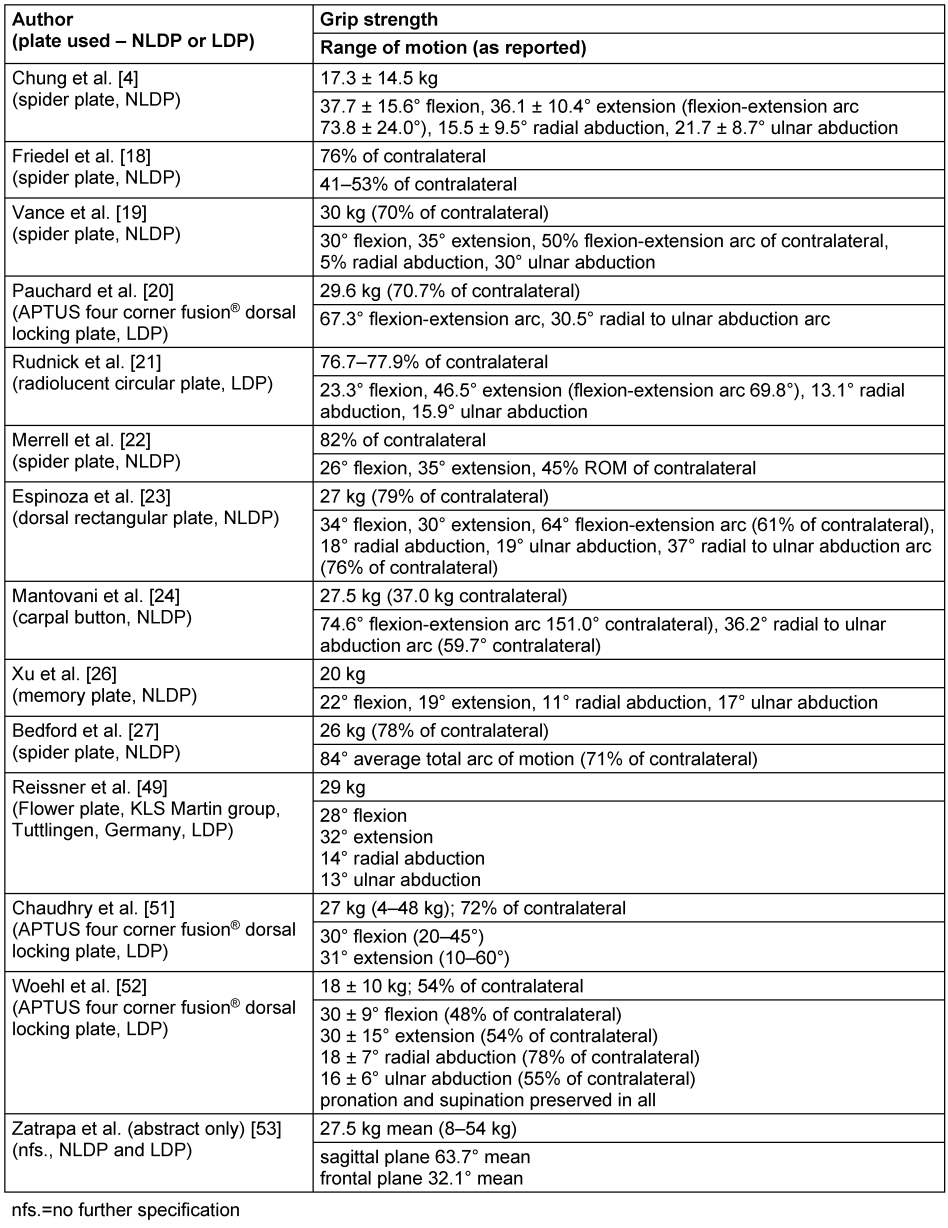
Comparison of results in grip strength and range of motion after four-corner arthrodesis via plate osteosynthesis in different working groups
